# Supplementary material for: Analysis of PPARγ Signaling Activity in Psoriasis
Source: Int J Mol Sci. 2021 Aug 10;22(16):8603. doi: 10.3390/ijms22168603 (PMC8395241; doi:10.3390/ijms22168603)
Supplement: Supplementary file 1 [file ijms-22-08603-s001.zip › Supplemental materials_Analysis of PPARg signaling activity in psoriasis/Pathway models/Models images and html files/Differentiation of psoriatic T cells/1799.html]

RUNX3


# Protein RUNX3

|  |  |
| --- | --- |
| URN | urn:agi-llid:864 |
| Total Entities | 0 |
| Connectivity | 1826 |
| Name | RUNX3 |
| Description | runt related transcription factor 3 |
| Notes | This gene encodes a member of the runt domain-containing family of transcription factors. A heterodimer of this protein and a beta subunit forms a complex that binds to the core DNA sequence 5'-PYGPYGGT-3' found in a number of enhancers and promoters, and can either activate or suppress transcription. It also interacts with other transcription factors. It functions as a tumor suppressor, and the gene is frequently deleted or transcriptionally silenced in cancer. Alternative splicing results in multiple transcript variants. [provided by RefSeq, Mar 2016] |
| Primary Cell Localization | Nucleus |
| Class | Transcription factor |

---

|  |  |
| --- | --- |
| Pathway | Proteins Involved in Melanoma |
|  | T-Cells Differentiation Block in Psoriasis |
|  | Th1-Cell Activation in Crohn's Disease |
|  | Proteins Involved in Hepatocellular Carcinoma |
|  | Hepatocellular Carcinoma |
|  | Growth Factor Signaling in Hepatocellular Carcinoma |
|  | Proteins Involved in Psoriatic Arthritis |
|  | Proteins Involved in Neuroblastoma |
|  | Proteins Involved in Endometriosis |
|  | Prostate Cancer |
|  | EZH2 Methyltransferase Role in Cancer |
|  | NOTCH -> Expression Targets in Thymus |
|  | PPAR Psoriasis |
|  | 1\_Differentiation of psoriatic T cells |
|  | 4\_1\_Polymorphisms associated with inflammatory bowel diseases |
|  | New Pathway |
|  | Differentiation of psoriatic T cells |

---

|  |  |
| --- | --- |
| Group | Genes Hypermethylated in Melanoma |
|  | Genes with Mutations Associated with Hepatocellular Carcinoma |
|  | Genes Hypermethylated in Glioma |

---

|  |  |
| --- | --- |
| MedScan ID | 864 |

---

|  |  |
| --- | --- |
| LocusLink ID | 864 |
|  | 12399 |
|  | 156726 |

---

|  |  |
| --- | --- |
| Alias | runt domain, alpha subunit 3 |
|  | FLJ34510 |
|  | core binding factor alpha 3 |
|  | acute myeloid leukemia-2 |
|  | Runt related transcription factor 3 |
|  | CBFA3 |
|  | runt-related transcription factor III |
|  | RP23-167O21.1 |
|  | core-binding factor subunit alpha-3 |
|  | acute myeloid leukemia 2 protein |
|  | transcription factor CBFA3 |
|  | core binding factor alpha 3 subunit |
|  | RP3-398I9.1 |
|  | RUNX3 |
|  | AML2 |
|  | polyomavirus enhancer-binding protein 2 alpha C subunit |
|  | transcription factor RUNX3 |
|  | core-binding factor, runt domain, alpha subunit III |
|  | CBF-alpha-3 |
|  | MGC16070 |
|  | acute myeloid leukemia 2 |
|  | SL3/AKV core-binding factor alpha C subunit |
|  | OTTHUMP00000003370 |
|  | runt domain, alpha subunit III |
|  | transcription factor AML2/CBFA3 |
|  | SL3-3 enhancer factor 1 alpha C subunit |
|  | PEBP2aC |
|  | acute myeloid leukemia gene-2 |
|  | oncogene AML-2 |
|  | OTTHUMP00000003371 |
|  | core-binding factor, runt domain, alpha subunit 3 |
|  | OTTMUSP00000042121 |
|  | acute myeloid leukemia gene 2 |
|  | PEA2 alpha C |
|  | PEBP2A3 |
|  | transcription factor AML2 |
|  | pebp2 alpha c |
|  | RUNX family transcription factor 3 |
|  | runt-related transcription factor 3 |
|  | PEA2-alpha C |
|  | PEBP2-alpha C |
|  | acute myeloid leukaemia gene 2 |

---

|  |  |
| --- | --- |
| GO ID | 0005524 |
|  | 0003700 |
|  | 0000981 |
|  | 0000977 |
|  | 0002062 |
|  | 0030097 |
|  | 0043371 |
|  | 0045786 |
|  | 0050680 |
|  | 0000122 |
|  | 0030182 |
|  | 0048935 |
|  | 0043378 |
|  | 0045893 |
|  | 0006468 |
|  | 0006355 |
|  | 0071559 |
|  | 0006366 |
|  | 0016513 |
|  | 0005737 |
|  | 0005829 |
|  | 0043231 |
|  | 0000790 |
|  | 0005730 |
|  | 0005654 |
|  | 0005634 |
|  | 0003677 |
|  | 0046332 |
|  | 0042826 |
|  | 0007411 |
|  | 0048469 |
|  | 0031069 |
|  | 0032609 |
|  | 0042789 |
|  | 0031175 |
|  | 2001238 |
|  | 0045944 |
|  | 0006357 |
|  | 0001503 |
|  | 0006351 |
|  | 0045595 |
|  | 0005794 |

---

|  |  |
| --- | --- |
| KEGG ID | hsa:864 |

---

|  |  |
| --- | --- |
| Organism | Homo sapiens {Organism urn:agi-taxid:9606} |
|  | Mus musculus {Organism urn:agi-taxid:10090} |
|  | Rattus norvegicus {Organism urn:agi-taxid:10116} |
|  | Mus musculus |
|  | Homo sapiens |
|  | Rattus norvegicus |

---

|  |  |
| --- | --- |
| Mouse chromosome position | 4 67.19 cM |

---

|  |  |
| --- | --- |
| OMIM ID | 600210 |

---

|  |  |
| --- | --- |
| Rat chromosome position | 5q36 |

---

|  |  |
| --- | --- |
| Hugo ID | 10473 |
|  | HGNC:10473 |

---

|  |  |
| --- | --- |
| Human chromosome position | 1p36.11 |
|  | 1p36 |

---

|  |  |
| --- | --- |
| Swiss-Prot Accession | A0A024RAH4 |
|  | Q13761 |
|  | Q13761.2 |
|  | Q3U1Q3 |
|  | Q64131.3 |
|  | Q91ZK1 |
|  | B1AJV5 |
|  | Q12969 |
|  | Q13760 |
|  | Q64131 |

---

|  |  |
| --- | --- |
| PIR ID | B55563 |
|  | S60078 |

---

|  |  |
| --- | --- |
| GenBank ID | NC\_000001 |
|  | NM\_001320672 |
|  | NP\_001307601 |
|  | NM\_001031680 |
|  | NP\_001026850 |
|  | NM\_004350 |
|  | NP\_004341 |
|  | XM\_005246024 |
|  | XP\_005246081 |
|  | XM\_011542351 |
|  | XP\_011540653 |
|  | AJ238394 |
|  | CAB42882 |
|  | AL023096 |
|  | AL445471 |
|  | CH471134 |
|  | EAW95147 |
|  | EAW95148 |
|  | EAW95149 |
|  | CQ834770 |
|  | CAH05600 |
|  | FB571463 |
|  | CAR95149 |
|  | FB571465 |
|  | CAR95150 |
|  | HI574042 |
|  | CBX47510 |
|  | HI574044 |
|  | CBX47511 |
|  | U14520 |
|  | AAA86465 |
|  | Z38104 |
|  | Z38108 |
|  | AF022726 |
|  | AAB80759 |
|  | AI375031 |
|  | AJ001432 |
|  | AK091829 |
|  | AK300056 |
|  | BAG61865 |
|  | AK301044 |
|  | BAG62657 |
|  | AK310945 |
|  | BC013362 |
|  | AAH13362 |
|  | DA935726 |
|  | EF491807 |
|  | EF491822 |
|  | X79550 |
|  | CAA56093 |
|  | Z35278 |
|  | CAA84541 |
|  | Q13761 |
|  | NC\_000070 |
|  | NM\_019732 |
|  | NP\_062706 |
|  | XM\_006538506 |
|  | XP\_006538569 |
|  | NT\_187024 |
|  | NM\_001369050 |
|  | AF169246 |
|  | AAD46381 |
|  | AF321443 |
|  | AAK11181 |
|  | AJ308681 |
|  | CAC41361 |
|  | AL731718 |
|  | CH466552 |
|  | EDL29993 |
|  | CU459015 |
|  | FJ026730 |
|  | S78518 |
|  | AAB34843 |
|  | AF085246 |
|  | AAC34127 |
|  | AF155880 |
|  | AAD38985 |
|  | AK053910 |
|  | BAC35587 |
|  | AK155436 |
|  | BAE33265 |
|  | AK155802 |
|  | BAE33440 |
|  | AK185845 |
|  | AK193548 |
|  | AK196105 |
|  | AK198691 |
|  | AK200435 |
|  | Q64131 |
|  | NC\_005104 |
|  | NM\_130425 |
|  | NP\_569109 |
|  | AC\_000073 |
|  | AABR07050147 |
|  | AABR07050148 |
|  | AAHX01040222 |
|  | AAHX01040223 |
|  | CH473968 |
|  | EDL80748 |
|  | AF421886 |
|  | AAL16092 |
|  | FQ230848 |
|  | NC\_018912 |
|  | AMYH02000534 |
|  | AMYH02000535 |
|  | XM\_006537058 |
|  | XP\_006537121 |
|  | AC\_000026 |
|  | AAHY01040740 |
|  | AAHY01040741 |
|  | BC168396 |
|  | AAI68396 |
|  | XM\_006711016 |
|  | XP\_006711079 |
|  | AC\_000133 |
|  | ABBA01059922 |
|  | ABBA01059923 |

---

|  |  |
| --- | --- |
| Swiss-Prot ID | RUNX3\_HUMAN |

---

|  |  |
| --- | --- |
| Cell Localization | Nucleus |
|  | Cytoplasm |

---

|  |  |
| --- | --- |
| Ensembl ID | ENSG00000020633 |
|  | ENSP00000382800.1 |
|  | ENST00000399916.5 |
|  | ENSP00000343477.3 |
|  | ENST00000338888.3 |
|  | ENSP00000308051.6 |
|  | ENST00000308873.10 |
|  | ENSMUSG00000070691 |
|  | ENSMUSP00000050353.7 |
|  | ENSMUST00000056977.13 |
|  | ENSRNOG00000054217 |
|  | ENSRNOP00000075140.1 |
|  | ENSRNOT00000086650.1 |
|  | ENSP00000382800 |
|  | ENST00000399916 |
|  | ENSP00000308051 |
|  | ENST00000308873 |
|  | ENSMUSP00000050353 |
|  | ENSMUST00000056977 |
|  | ENSRNOP00000075140 |
|  | ENSRNOT00000086650 |
|  | ENSRNOG00000018058 |
|  | ENSRNOP00000045395 |
|  | ENSRNOT00000041474 |

---

|  |  |
| --- | --- |
| MGI ID | MGI:102672 |

---

|  |  |
| --- | --- |
| RGD ID | 620082 |

---

|  |  |
| --- | --- |
| Unigene ID | Hs.170019 |
|  | Mm.378894 |
|  | Rn.34220 |

---

|  |  |
| --- | --- |
| Homologene ID | 37914 |

---

|  |  |
| --- | --- |
| Shape | O-vertex |

---
